# Increased Risk for Maternal Anxiety during the COVID-19 Outbreak in Brazil among Pregnant Women without Comorbidities

**DOI:** 10.1055/s-0041-1740234

**Published:** 2021-12-21

**Authors:** Roseli Mieko Yamamoto Nomura, Ana Carla Franco Ubinha, Isabela de Paula Tavares, Maria Laura Costa, Maria Lucia da Rocha Opperman, Marianna Facchinetti Brock, Alberto Trapani, Lia Cruz Vaz da Costa Damásio, Nadia Stella Viegas Reis, Vera Therezinha Medeiros Borges, Alberto Carlos Moreno Zaconeta, Ana Cristina Pinheiro Fernandes de Araujo, Rodrigo Ruano

**Affiliations:** 1 Department of Obstetrics, Escola Paulista de Medicina, Universidade Federal de São Paulo, São Paulo, SP, Brazil; 2 Department of Obstetrics and Gynecology, Universidade de Campinas, Campinas, SP, Brazil; 3 Department of Gynecology and Obstetrics, Hospital de Clínicas, Faculdade de Medicina, Universidade Federal do Rio Grande do Sul, Porto Alegre, RS, Brazil; 4 Department of Obstetrics and Gynecology, University of Amazonas State, Manaus, AM, Brazil; 5 Women's Health Care Unit, Hospital Universitário, Universidade Federal de Santa Catarina, Florianópolis, SC, Brazil; 6 Department of Gynecology and Obstetrics, Universidade Federal do Piaui, Teresina, PI, Brazil; 7 Department of Gynecology and Obstetrics, Hospital Universitário da Faculdade de Medicina Maria Aparecida Pedrossian, Universidade Federal do Mato Grosso do Sul, Campo Grande, MS, Brazil; 8 Department Obstetrics and Gynecology, Faculdade de Medicina de Botucatu, Universidade Estadual Paulista, Botucatu, SP, Brazil; 9 Department of Gynecology and Obstetrics, Faculty of Medicine, Hospital Universitário de Brasília, Universidade de Brasília, Brasília, DF, Brazil; 10 Department of Gynecology and Obstetrics, Maternidade Januário Cicco, Universidade Federal do Rio Grande do Norte, Natal, RN, Brazil; 11 Maternal-Fetal Medicine Division, Department of Obstetrics and Gynecology, Mayo Clinic College of Medicine, Rochester, MN, United States

**Keywords:** pregnancy, maternal anxiety, childbirth, postpartum, questionnaires, breastfeeding, coronavirus disease 2019, pandemic, gravidez, ansiedade materna, parto, pós-parto, questionários, amamentação, infecção por coronavírus 2019, pandemia

## Abstract

**Objective** To study maternal anxiety in pregnant women without comorbidities in the context of the COVID-19 outbreak in Brazil and to study maternal knowledge and concerns about the pandemic.

**Methods** This is a secondary analysis from a national multicenter cross-sectional study performed in 10 cities, from June to August, 2020, in Brazil. Interviewed postpartum women, without medical or obstetrical comorbidities, were included in the present subanalysis. A structured questionnaire and the Beck Anxiety Inventory (BAI) were applied.

**Results** Out of the 1,662 women, 763 (45.9%) met the criteria for the current analysis and 16.1% presented with moderate and 11.5% with severe maternal anxiety. Moderate or severe maternal anxiety was associated with high school education (odds ratio [OR]:1.58; 95% confidence interval [CI]:1.04–2.40). The protective factor was cohabiting with a partner (OR: 0.46; 95%CI: 0.29–0.73). There was a positive correlation between the total BAI score and receiving information about care in the pandemic (r _partial_ 0.15; *p* < 0.001); concern about vertical transmission of COVID-19 (r _partial_ 0.10; *p* = 0.01); receiving information about breastfeeding (r _partial_ 0.08; *p* = 0.03); concerns about prenatal care (r _partial_ 0.10; *p* = 0.01), and concerns about the baby contracting COVID-19 (r _partial_ 0.11; *p* = 0.004). The correlation was negative in the following aspects: self-confidence in protecting from COVID-19 (r _partial_ 0.08; *p* = 0.04), having learned (r _partial_ 0.09; *p* = 0.01) and self-confidence in breastfeeding (r _partial_ 0.22; *p* < 0.001) in the context of the pandemic.

**Conclusion** The anxiety of pregnant women without medical or obstetrical comorbidities was associated to high school educational level and not living with a partner during the COVID-19 pandemic. Self-confidence in protecting against COVID-19 and knowledge about breastfeeding care during the pandemic reduced maternal anxiety.

## Resumo

**Objetivo** Estudar a ansiedade materna em gestantes sem comorbidades no contexto do surto de COVID-19 no Brasil e estudar o conhecimento e as preocupações maternas sobre a pandemia.

**Métodos** Trata-se de análise secundária de um estudo transversal multicêntrico nacional realizado em 10 cidades, de junho a agosto de 2020, no Brasil. Mulheres no pós-parto entrevistadas, sem comorbidades médicas ou obstétricas, foram incluídas nesta subanálise. Foram aplicados um questionário estruturado e o Inventário de Ansiedade de Beck (BAI, na sigla em inglês).

**Resultados** Das 1.662 mulheres, 763 (45,9%) atenderam aos critérios da análise atual e 16,1% apresentaram ansiedade materna moderada e 11,5% ansiedade materna grave. A ansiedade materna moderada ou grave foi associada à escolaridade no ensino médio (odds ratio [OR]: 1,58; intervalo de confiança [IC] 95%: 1,04–2,40). O fator protetor foi coabitar com companheiro (OR: 0,46; IC95%: 0,29–0,73). Houve correlação positiva entre a pontuação total do BAI e o recebimento de informações sobre cuidados na pandemia (r _parcial_ 0,15; *p* < 0,001); preocupação com a transmissão vertical de COVID-19 (r _parcial_ 0,10; *p* = 0,01); receber informações sobre amamentação (r _parcial_ 0,08; *p* = 0,03); preocupações sobre cuidados pré-natais (r _parcial_ 0,10; *p* = 0,01) e preocupações sobre o bebê contrair COVID-19 (r _parcial_ 0,11; *p* = 0,004). A correlação foi negativa com os seguintes aspectos: ter autoconfiança para se proteger (r _parcial_ 0,08; *p* = 0,04), aprender (r _parcial_ 0,09; *p* = 0,01) e ter autoconfiança para amamentar (r _parcial_ 0,22; *p* < 0,001) no contexto da pandemia.

**Conclusão** A ansiedade de gestantes sem comorbidades médicas ou obstétricas esteve associada à escolaridade no ensino médio e não morar com companheiro durante a pandemia de COVID-19. A autoconfiança na proteção contra COVID-19 e o conhecimento sobre os cuidados com a amamentação durante a pandemia reduziram a ansiedade materna.

## Introduction

Coronavirus disease 2019 (COVID-19) is a respiratory infection caused by the SARS-CoV-2 coronavirus, which is potentially serious and highly transmissible. 1 The first case was identified in December 2019 in the city of Wuhan, China, 2 and the infection quickly reached global proportions and was declared a pandemic by the World Health Organization (WHO) on March 11, 2020. 3

In Brazil, the first case of COVID-19 was detected on February 26, 2020, in São Paulo, and due to the rapid growth in the number of occurrences in Brazil, on May 22 the WHO declared South America as the epicenter of the pandemic. Brazil is among the countries with the highest number of cases and deaths from COVID-19. The mortality of hospitalized patients is high even in those < 60 years old, mainly due to regional disparities and delays within the health system. 4

Since the beginning of the pandemic, it has been observed that the elderly and people with comorbidities are vulnerable to the severe form of COVID-19. 1 However, other groups have been associated with an increased severity of the disease, including pregnant and postpartum women. Even among healthy pregnant women without disease or any identified previous medical morbidity, COVID-19 can progress to severe maternal morbidity and maternal death. In Brazil, COVID-19 has been associated with increased maternal deaths, with a substantial proportion of these deaths occurring without intensive care or respiratory support, suggesting delays in the provision of health care. Takemoto et al. 5 found a case fatality rate of 12.7% among women with severe acute respiratory syndrome due to COVID-19 during pregnancy and the postpartum period, using the national surveillance system data on severe respiratory diseases.

In this critical scenario, a national online survey applied to the Brazilian population during the pandemic found a high prevalence of depression (61%) and anxiety (44%). 6 Pregnant women may be vulnerable to anxiety due to increased concerns about childbirth and the risks of vertical transmission. The effects of maternal infection on the fetus and on the newborn are a source of concern and insecurity, 7 since COVID-19 during pregnancy is associated with an increased risk of adverse maternal and perinatal outcomes. 8

Results from the main study showed the impact of the COVID-19 pandemic on maternal anxiety in Brazil. 9 Our study found moderate or severe anxiety in 23.4% of the women at the end of pregnancy. The present secondary analysis addressed a subgroup of interviewed women who had no comorbidity in pregnancy and childbirth. The aim was to verify the presence of maternal anxiety in pregnant women without medical or obstetrical comorbidities in the context of the COVID-19 outbreak and to analyze the association with maternal knowledge and concerns about the pandemic.

## Methods

A multicenter cross-sectional survey design including two questionnaires was performed with data collected in 10 cities in Brazil. This is a secondary data analysis from a larger study on anxiety during pregnancy. The study protocol was approved by the Brazilian National Ethics Committee – CONEP (CAAE N° 31190120.6.1001.5505) and by each local Research Ethics Committee where the data was collected. A written informed consent form was signed by all included participants.

Women who delivered in the 10 public university hospitals were recruited from June 1, 2020, to August 31, 2020. Enrolment took place for a period of 60 consecutive days at each center. All hospitals were linked to a federal or state public university located in 10 cities: Manaus, state of Amazonas, Natal, state of Rio Grande do Norte, Teresina, state of Piaui, São Paulo, state of São Paulo, Campinas, state of São Paulo, Botucatu, state of São Paulo, Florianópolis, state of Santa Catarina, Porto Alegre, state of Rio Grande do Sul, Campo Grande, state of Mato Grosso do Sul, and Brasília, Federal District. Each university hospital had a local coordinator and trained medical residents who were involved in applying the questionnaires.

For the present analysis, we considered postpartum women without medical or obstetrical comorbidities> 18 years old; childbirth birth > 36 weeks of gestation; single and alive newborn without malformations; no clinical suspicion or current diagnosis of COVID-19; absence of psychiatric or mental disorder; and in good clinical condition. The women were interviewed after childbirth and before hospital discharge. They were asked to complete a sociodemographic questionnaire with questions that included maternal age, parity, educational level, marital status, habits (smoking and consumption of alcohol and illicit drugs), companionship during labor, gestational age at birth, mode of delivery, birthweight, 5 ^-^ minute Apgar score, history of COVID-19 during pregnancy, and history of COVID-19 in the family.

A face-to-face interview was conducted using a questionnaire with statements addressing their knowledge and concerns about the COVID-19 pandemic, including information received on prenatal care and instructions for childbirth and the postpartum, including breastfeeding. This questionnaire comprised four domains: general knowledge and preventive care (4 items), prenatal concerns (4 items), cautions and fears during childbirth (5 items), care and concerns about the newborn (5 items). Each item received a 5-point Likert response ranging from 1 to 5 (strongly disagree, partially agree, indifferent, partially agree, and fully agree). This questionnaire was specifically designed for the present study.

The Beck Anxiety Inventory (BAI) was used to measure maternal anxiety. The questions were answered following instructions to report symptoms of the last 7 days before delivery. The BAI consists of a 21-item self-reported questionnaire for assessing anxiety level. Each item describes a common symptom of anxiety and is rated on a 4-point Likert scale ranging from 0 (not at all) to 3 (severe). The respondent was asked to rate each symptom and then the total score was calculated (0–63). A high overall score indicates a high level of anxiety. Anxiety levels are defined according to the total score as follows: minimal anxiety (0–7), mild anxiety (8–15), moderate anxiety (16–25), and severe anxiety (26–63). 10 A validated Brazilian Portuguese version of the BAI was used in the present study. 11

Data were analyzed using MedCalc Statistical Software version 19.5.3 (MedCalc Software Ltd, Ostend, Belgium). Descriptive statistics are presented as mean and standard deviation (SD), median (IQR), or frequency and percentage (%). The associations of categorical variables with binary outcomes were analyzed using the chi-squared test or the Fisher exact test when appropriate. The Mann-Whitney U test was applied to continuous variables with nonparametric distribution. The analyses were adjusted using logistic regression for potential confounders: maternal age, nulliparity, race, educational level, marital status, religious belief, smoking, alcohol consumption, and geographical location. Correlation analysis was performed by the Spearman rank test and multiple regression with enter procedure was used to identify independent variables correlated with the BAI total score. Statistical significance was set at *p* < 0.05.

## Results

During the 3-month period of the present study, 1,683 eligible women were invited to participate in the study and 21 refused. Out of 1,662 women interviewed, 763 were included in the present subanalysis since they did not have any disease or medical comorbidity. Characteristics of the studied population and the overall results of the BAI score are shown in Table 1 . Most women were non-white and living with a partner. The BAI total score in late pregnancy indicates that 16.1% presented moderate and 11.5% severe anxiety.

**Table 1:** Sociodemographic and obstetric characteristics, perinatal outcomes, and maternal anxiety assessed by the Beck Anxiety Inventory (BAI) in pregnant women without medical or obstetrical comorbidities during the COVID-19 outbreak in Brazil ( *n* = 763)

Characteristics	Results	
Maternal age, years old, mean, SD	27.1	(6.3)
Parity 0	311	40.8
Maternal race		
White	233	30.5%
Mixed	443	58.1%
Black	74	9.7%
Asian or Brazilian Indian	13	1.7%
Cohabiting / married	667	87.4%
Educational level		
Incomplete elementary school	4	0.5%
Elementary school	186	24.4%
High school	455	59.6%
College/University	118	15.5%
Religion		
Evangelical	276	36.2%
Catholic	263	34.5%
Others	36	4.7%
Without religious belief	188	24.6%
Smoking	36	4.7%
Alcohol consumption	26	3.4%
Illicit drugs consumption	5	0.7%
Mode of delivery		
Vaginal	451	59.1%
Cesarean	298	39.1%
Forceps / vacuum	14	1.9%
Companionship in labor	648	84.9%
Gestational age at birth, weeks, mean, SD	39.3	(1.2)
Birth weight, g, mean, SD		
Low birthweight (< 2,500g)	28	3.7%
Macrosomia (> 4,000g)	63	8.3%
5-minute Apgar < 7	19	2.5%
COVID-19 during pregnancy	13	1.7%
COVID-19 in the family	31	4.1%
Maternal anxiety		
Minimal	344	45.1%
Mild	208	27.3%
Moderate	123	16.1%
Severe	88	11.5%
BAI, total score		
mean, SD	11.4	(10.5)
median (95%CI)	8.0	(8.0–9.0)

Abbreviations: BAI, Beck Anxiety Inventory; CI, confidence interval; SD, standard deviation.

In the period of the present study, maternal anxiety according to each geographic region is presented in Table 2 . The BAI total score was significantly higher in women interviewed in the Central West, in the South and in the Southeast than those in the North and in the Northeast regions. The Northeast exhibited the lowest prevalence of maternal anxiety of all regions. The Central West region had the highest proportion of moderate or severe maternal anxiety.

**Table 2:** Maternal anxiety of pregnant women without medical or obstetrical comorbidities according to geographic regions during the COVID-19 outbreak in Brazil

	Total		Geographic region									
			Central West		North		Northeast		South		Southeast	
			( *n* = 150)		( *n* = 161)		( *n =* 106)		( *n* = 181)		( *n* = 165)	
Maternal anxiety (BAI)												
Minimal (0–7)	344	(45,1%)	63	(42.0)	82	(50.9)	61	(57.5)	75	(41.4)	63	(38.2)
Mild (8–15)	208	(27,3%)	41	(27.3)	43	(26.7)	25	(23.6)	48	(26.5)	51	(30.9)
Moderate (16–25)	123	(16,1%)	29	(19.3)	20	(12.4)	12	(11.3)	34	(18.8)	28	(17.0)
Severe (26–63)	88	(11,5%)	17	(11.3)	16	(9.9)	8	(7.5)	24	(13.3)	23	(13.9)
BAI score												
Mean (SD)	11.4	(10.5)	12.1	(10.4)	10.2	(11.6)	8.4	(8.4)	12.4	(10.7)	12.6	(10.3)
Median (95%CI)*	8.0	(8.0–9.0)	10.0	(7.5–11.5)	5.0	(4.6–9.0)	7.0	(5.0–8.0)	10.0	(8.0–12.0)	10.0	(8.0–12.0)

Abbreviations: BAI, Beck Anxiety Inventory; CI, confidence interval; SD, standard deviation.

* Kruskal-Wallis test *p* <0.001. Post-hoc analysis (Conover): Central West different from North and Northeast, *p* < 0.05; North different from Central West, South, and Southeast, *p* < 0.05; Northeast different from Central West, South, and Southeast, *p* < 0.05; South different from North and Northeast, *p* < 0.05; Southeast different from North and Northeast, *p* < 0.05.

The crude and adjusted analysis for confounding factors of moderate or severe maternal anxiety is presented in Table 3 . The results showed the variable 'cohabiting with a partner' (adjusted odds ratio [aOR]: 0.46. 95% confidence interval [CI]: 0.29–0.73) as a protective factor for maternal anxiety and 'high school educational level' (aOR: 1.58; 95%CI: 1.04–2.40) as an independent factor significantly associated with moderate or severe maternal anxiety at the end of pregnancy.

**Table 3:** Characteristics of women and geographic region in Brazil according to moderate or severe maternal anxiety as assessed by the Beck Anxiety Inventory during the COVID-19 outbreak

Characteristic	Maternal anxiety				
	Minimum or mild	Moderate or severe ( *n* = 211)	*p-value* ^a^	aOR (95%CI) ^b^	*p-value* ^b^
	( *n* = 552)				
Age, years old, median, AVR	26 (385.9)	26 (371.7)	0.426	–	0.896
Parity 0	212 (38.4)	99 (46.9)	0.032	–	0.133
Maternal race					
White	154 (27.9)	79 (37.4) 33.9	0.011 ^c^	–	0.137
Nonwhite	398 (72.1)	132 (62.6) 24.9			
Cohabiting / married	495 (89.7)	172 (81.5)	0.002	0.46 (0.29–0.73)	0.001
Educational level					
High school	305 (55.3)	140 (66.4)	0.007	1.58 (1.04–2.40)	0.030
College/University	89 (16.1)	29 (24.6)	0.484	–	0.985
No religious belief	136 (24.6)	52 (24.6)	0.998	–	0.430
Smoking	21 (3.8)	15 (7.1)	0.054	–	0.154
Alcohol consumption	17 (3.1)	9 (4.3)	0.420	–	0.290
COVID-19 during pregnancy	8 (1.4)	5 (2.4)	0.380	–	0.433
COVID-19 in the family	20 (3.6)	11 (5.2)	0.320	–	0.473
Geographic location					
Central West	104 (18.8)	46 (21.8)	–	–	–
North	125 (22.6)	36 (17.1)	0.125	–	0.139
Northeast	86 (15.6)	20 (9.5)	0.048	–	0,064
South	123 (22.3)	58 (27.5)	0.881	–	0.910
Southeast	114 (20.7)	51 (24.2)	0.940	–	0.821

Abbreviations: aOR: adjusted odds ratio; AVR: average rank; CI: confidence interval.

Data are presented as median (average rank) or number (percentage); ^a^ Chi-squared test; ^b^ Logistic regression to identify independent variables; ^c^ White versus nonwhite; ^d^ Black versus *.* nonblack.

The correlation analysis of the scores of questionnaire items on the knowledge and concerns of the mother about COVID-19 with the BAI score are presented in Table 4 . After adjustment by multiple regression, there was a positive and significant correlation between the total BAI score and the items referring to: being informed about care in the pandemic (r _partial_ 0.15; *p* < 0.001); concern about vertical transmission (r _partial_ 0.10; *p* = 0.01); be guided on breastfeeding (r _partial_ 0.08; *p* = 0.03); concern about difficulties in prenatal care during the pandemic (r _partial_ 0.10; *p* = 0.01), and concern about the baby contracting COVID-19 (r _partial_ 0.11; *p* = 0.004). The following variables were protective for maternal anxiety: self-confidence in protecting from COVID-19 (r _partial_ - 0.08; *p* = 0.04), having learned (r _partial_ - 0.09; *p* = 0.01) and having self-confidence in breastfeeding (r _partial_ - 0.22; *p* < 0.001) in the context of the pandemic.

**Table 4:** Rank correlation analysis between maternal knowledge and concern items scores and Beck Anxiety Inventory total score

Questionnaire items	Rho	*p-value* a	Coefficient	Standard error	t	*p-value* ^b^	r _partial_
Knowledge and preventive care						–	–
I am afraid about getting COVID-19.	−0.033	0.367	−0.409	0.323	−1.27	0.206	−0.046
I know the signs and symptoms of COVID-19.	−0.033	0.364	−0.410	0.307	−1.34	0.182	−0.049
I received information about care in the pandemic.	0.104	0.004	1.350	0.334	4.05	<0.001	0.147
I feel confident in protecting myself from COVID-19.	−0.073	0.043	−0.630	0.298	−2.11	0.035	−0.077
Prenatal concerns							
I was worried about COVID-19 affecting my baby during pregnancy.	0.074	0.040	1.059	0.382	2.77	0.006	0.101
I was instructed on caring for the newborn.	0.025	0.495	−0.362	0.313	−1.16	0.247	−0.043
I was guided on breastfeeding during COVID-19.	0.093	0.010	0.630	0.288	2.18	0.029	0.080
I was worried about prenatal difficulties.	0.067	0.063	0.796	0.300	2.65	0.008	0.097
Cautions and fears during childbirth							
I received guidance on childbirth care due to COVID-19.	0.031	0.395	0.134	0.237	0.56	0.573	0.021
My companion was afraid of COVID-19 at delivery.	0.076	0.036	0.171	0.253	0.68	0.499	0.025
I was worried about giving birth at the hospital.	−0.020	0.582	−0.107	0.283	−0.38	0.706	−0.014
I was afraid to be without a companion at childbirth.	0.030	0.412	0.181	0.319	0.57	0.572	0.021
I was worried that childbirth care might be compromised due to COVID-19.	−0.030	0.406	−0.113	0.272	−0.42	0.678	−0.015
Care and concerns about the newborn							
I learned how to breastfeed due to COVID-19.	−0.050	0.164	−0.614	0.245	−2.51	0.012	−0.092
I feel confident to breastfeed despite COVID-19.	−0.161	<0.001	−2.369	0.382	−6.20	<0.001	−0.222
I am worried about having COVID-19.	0.009	0.798	−0.637	0.360	−1.77	0.077	−0.065
I am worried that my baby has COVID-19.	0.011	0.757	−0.321	0.301	−1.07	0.287	−0.039
I am worried about my baby having COVID-19 after birth.	0.093	0.010	1.208	0.413	2.92	0.004	0.107
(Constant)	–	–	13.166	–	–	–	–

a Spearman correlation; ^b^ Multiple regression with enter procedure to identify independent variables.

## Discussion

This is the first multicenter study in Brazil to investigate anxiety of pregnant women without medical or obstetrical comorbidities during the COVID-19 outbreak by face-to-face interviews. Our study found moderate or severe anxiety in 27.6% of the healthy women at the end of pregnancy. Moderate or severe maternal anxiety was associated with high school educational level and not living with a partner. Women who were better informed during the pandemic and who demonstrated concerns about prenatal care, vertical transmission, or about the baby contracting COVID-19 presented increased maternal anxiety evaluated by the BAI total score. Self-confidence in protecting against COVID-19 and knowledge about breastfeeding care during the pandemic reduced maternal anxiety.

The COVID-19 pandemic has led to adverse mental health consequences in the general population. 12 Multiple COVID-19-related factors should be considered, such as perceived risk and concerns about infection, full and partial lockdowns, and social restriction measures. 13 Studies have examined general anxiety related to worries about the self and the baby during COVID-19 pandemic. 9 14 15 Matvienko-Sikar et al. 16 found significant decreases in the perceived social support from all sources by pregnant women during the COVID-19 pandemic, and a nonsignificant increase in stress. Our study found that anxiety was decreased in those living with a partner, which is an important factor of social support in our culture.

Several patient groups were found to be more vulnerable to COVID-19 during the pandemic, and pregnant women are at a higher risk of death in Brazil. 17 Maternal and fetal effects, as well as the best management of COVID-19 in pregnancy, have not been completely elucidated. 18 These uncertainties and changes may be the main aspects related to maternal anxiety during the pandemic.

Women are less concerned about their own health; nevertheless, many of them were significantly anxious. In Wuhan, China, the COVID-19 outbreak increased the anxiety of pregnant women and affected their decision-making regarding prenatal care schedules or timing of childbirth, mode of delivery, and infant feeding. 19 Wu et al. 20 reported depressive symptoms in 29.6% of pregnant women after the declaration of an epidemic. In Belgium, an online survey during the lockdown period revealed that 14% of pregnant and breastfeeding women met the criteria for high anxiety. 21 Interventions targeting maternal stress and isolation, such as effective communication and psychological support, should be offered to decrease these mental health effects.

In the present study, data were collected from all geographic regions in Brazil during the same period. Maternal anxiety was more prevalent and more severe in the Central West and in the South regions during the studied period. At that time, COVID-19 was receding in the North and in the Northeast and increasing in the South and in the Central West. This may have influenced the prevalence of maternal anxiety. The notifications of increased numbers of deaths could potentially impact maternal mental health, and the risk of anxiety disorders may have increased as a result. Liu et al. 19 found that more women felt anxious in Wuhan than in Chongqing, because the first city was more affected by COVID-19. Brazil has great disparities among regions, not only in COVID-19 mortality cases, but in other health indicators and in social, cultural, and economic characteristics.

Another source of concern is not being able to reach the prenatal care appointments. Even though uninterrupted prenatal care was provided during the pandemic, the frequency of appointments diminished, and the same occurred with exams and ultrasounds. The uncertainty about the best treatment and clinical management of patients with COVID-19 may also affect the mind of pregnant women. 22 Uncertain prognosis, social restrictions, economic financial losses, decline in quality of life, and conflicting messages from government authorities are additional stressors and, possibly, trigger mental health crises.

An online survey in Belgium revealed higher levels of overall anxiety among pregnant women, 8.4% of whom had moderate and 5.2% had severe anxiety. 21 We found 16.1% cases of moderate anxiety and 11.5% cases of severe anxiety. These differences may be related to the fact that we conducted face-to-face, not online, interviews,. We also found that high school educational level and not living with a partner were associated with higher scores on the total BAI, indicating that social support should be improved during antenatal care. It is important for women to have adequate support, which includes health care workers and companions during labor and childbirth, to improve their mental well-being. Specific interventions that aim to reduce COVID-19 stress may help to reduce overall stress levels in pregnant women during the pandemic. 23

The strength of the present study lies in the inclusion of 10 cities in all geographic regions in Brazil, most of which are state capitals. Additionally, the women were interviewed face-to-face, not through online forms or phone calls. All the interviews were performed by trained doctors who were available to answer questions and minimize concerns. Our study has the limitation of including only women from receiving care from the public sector in university hospitals. Another limitation is that the emergence of COVID-19 was different in each geographic region, which present organizational differences in health systems in cities, and this may have influenced the quality of health care.

## Conclusion

Anxiety of pregnant women without medical or obstetrical comorbidities was related to high school educational level and not living with a partner during the COVID-19 pandemic. Women who were better informed during the pandemic and who demonstrated concerns about prenatal care, vertical transmission, or about the baby contracting COVID-19 presented increased maternal anxiety. Self-confidence in protecting against COVID-19 and knowledge about breastfeeding care during the pandemic reduced maternal anxiety.
